# Unlocking the potential of supported liquid phase catalysts with supercritical fluids: low temperature continuous flow catalysis with integrated product separation

**DOI:** 10.1098/rsta.2015.0005

**Published:** 2015-12-28

**Authors:** Giancarlo Franciò, Ulrich Hintermair, Walter Leitner

**Affiliations:** 1 Institut für Technische Chemie und Makromolekulare Chemie, RWTH Aachen University, Worringerweg 2, Aachen 52074, Germany; 2 Centre for Sustainable Chemical Technologies, University of Bath, Claverton Down, Bath BA2 7AY, UK; 3 Max-Planck-Institut für Kohlenforschung, Kaiser-Wilhelm-Platz 1, Mülheim an der Ruhr 45470, Germany

**Keywords:** supercritical CO_2_, homogeneous catalysis, supported liquid phases, ionic liquids, catalyst recycling, continuous-flow processing

## Abstract

Solution-phase catalysis using molecular transition metal complexes is an extremely powerful tool for chemical synthesis and a key technology for sustainable manufacturing. However, as the reaction complexity and thermal sensitivity of the catalytic system increase, engineering challenges associated with product separation and catalyst recovery can override the value of the product. This persistent downstream issue often renders industrial exploitation of homogeneous catalysis uneconomical despite impressive batch performance of the catalyst. In this regard, continuous-flow systems that allow steady-state homogeneous turnover in a stationary liquid phase while at the same time effecting integrated product separation at mild process temperatures represent a particularly attractive scenario. While continuous-flow processing is a standard procedure for large volume manufacturing, capitalizing on its potential in the realm of the molecular complexity of organic synthesis is still an emerging area that requires innovative solutions. Here we highlight some recent developments which have succeeded in realizing such systems by the combination of near- and supercritical fluids with homogeneous catalysts in supported liquid phases. The cases discussed exemplify how all three levels of continuous-flow homogeneous catalysis (catalyst system, separation strategy, process scheme) must be matched to locate viable process conditions.

## 1. Introduction

Molecularly defined transition metal complexes [1] are able to catalyse a large variety of reductive, redox-neutral and oxidative processes, often with high rates and astonishing selectivities [2] including asymmetric control [3]. Thus, this technology can be considered as a pillar of green chemistry as it allows to selectively and atom efficiently produce compounds and materials [4]. Application of this technology on production scale is, however, often hampered by the homogeneous nature and intrinsic sensitivity of the catalysts; many systems only perform well in certain concentration regimes, are thermally unstable and/or deactivate when exposed to air, moisture and certain solvents [5]. One processing strategy is to maximize their productivity (i.e. turnover number, TON) in an intensified batch reaction, and then sacrifice the catalyst during a product-targeted workup procedure [6]. This strategy can be a viable option if the balance of catalyst cost and productivity versus product value yields positive process economics [7]. A more sustainable and more general approach is to prevent catalyst deactivation throughout the entire process, and seek its recovery and re-use [8]. This option promises higher material efficiencies [9] over the batch-wise ‘run until you die’ approach, but imposes significant engineering challenges on the process. Some existing large-scale applications of homogeneous catalysis, such as propene hydroformylation to butyraldehyde, methanol carbonylation to acetic acid or propene methoxycarbonylation to methylmetacrylate [10,11], have shown how recycling and continuous processing can be achieved with highly productive and robust systems that can be separated from the reaction products due to distinct volatility or solubility properties. To unlock this potential also for transformations of larger and more complex molecules for fine chemical and pharmaceutical production, new technologies need to be developed to overcome the more delicate catalyst-product separation challenge without endangering the thermally sensitive catalysts and products in these cases.

**Figure 1.**
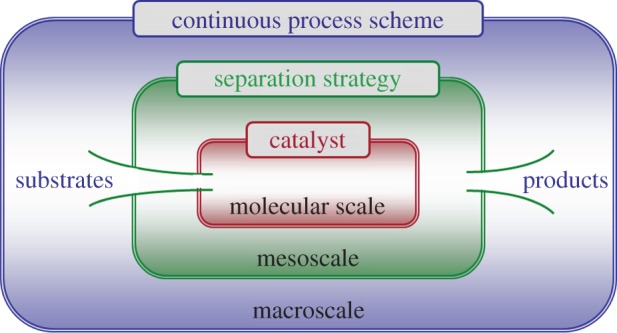
Conceptual levels of integrated continuous-flow homogeneous catalysis [12].

Generally, an ideal solution would be to introduce a separation strategy that is gentle enough to prevent catalyst deactivation yet effective enough to be directly applicable to turnover conditions. If then a compatible process scheme can be designed around this, an integrated continuous-flow process may be realized (figure 1) [12,13]. This not only resolves the issue of catalyst recovery but also affords a number of well-known engineering advantages, including intensified space–time yields (STYs), reduced waste production and enhanced process control through more effective heat management and automation [14,15]. This challenge needs to be addressed in a multi-scale approach considering all conceptual levels from the molecular catalyst to the process [12].

**Figure 2.**
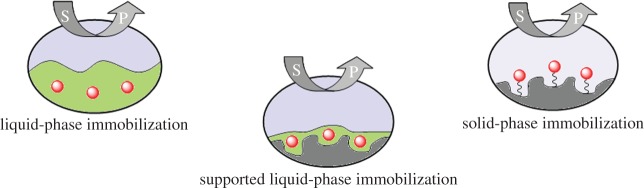
Permanentseparation strategies based on phase boundaries to discriminate reaction products from the molecular catalyst (red spheres) for continuous processing.

Multiphasic systems consisting of a molecular catalyst in a product-separable fluid phase or on the surface of a solid support proved to be viable strategies in this regard [8]. Phase boundaries are convenient separation strategies to effect product-catalyst discrimination at mild temperatures, and may be introduced by e.g. inorganic oxide materials, organic polymers, water, designer solvents such as fluorous phases or ionic liquids (ILs), and supercritical fluids (SCFs) with tuneable solvent properties [16]. However, most multiphasic systems seeking to bridge the gap between homogeneous and heterogeneous catalysis [17,18], also combine some of their respective disadvantages at the same time. For instance, when using molecular catalysts in multiphasic systems, the accessibility, characterizability and tuneability of the once homogeneous catalysts are compromised by various degrees for the sake of their retention in continuous operation [12]. A particularly promising approach to reconcile accessibility and structural variation of immobilized molecular catalysts with effective retention is to use them in supported liquid phases (SLPs): dispersion of a concentrated catalyst solution on the surface of a porous support combines the respective advantages of liquid and solid phase covalent immobilization (figure 2 and table 1) [19]. As will be shown in this article, the combination of this immobilization strategy with supercritical carbon dioxide (scCO_2_) as transport medium for continuous-flow applications is particularly attractive to establish practically ‘solvent-free’ processes for asymmetric synthesis.

**Table 1.** Key characteristics of the immobilization approaches shown in figure 2 (STY=space–time yield).

immobilization approach	homogeneous nature of catalyst	synthetic catalyst modification	transport limitation	engineering of continuous process	reactor volume (process efficiency)
liquid	preserved	none to	liquid diffusion	extensive	large
phase		moderate	(Hatta [19])		(low STYs)
solid phase	restricted	substantial	pore diffusion	straightforward	small
(covalent binding)			(Thiele [19])		(high STYs)
supported liquid	preserved	none to	adjustable through	straightforward	small
phase		moderate	loading (vide infra)		(high STYs)

On the molecular level, SLPs represent a particularly gentle immobilization technique because it immobilizes the solvent and not the catalyst. Therefore, modification of the catalyst itself is not required in most cases (e.g. water or fluorus phase will require tagged ligands for ensuring sufficient solubility in the catalyst phase) and similar mechanisms and kinetics as in bulk solution prevail in the SLP. It is also beneficial to the macroscale process scheme because the bulk properties of the catalyst material are dominated by the solid support, and thus the solvent is kept entirely on the mesoscale where it permits homogeneous catalytic turnover [12]. Challenges include compatibility and fine-tuning of the components (support–liquid–catalyst; see §4b) and the location of process parameters that effectively avoid leaching (see §4e). In the following, we provide a brief overview of the development of SLP catalysts and highlight some characteristics and applications, focusing on promising recent developments based on supported IL phase catalysts with near- and SCFs. Many of these catalytic systems have stable long-term activity, high selectivity and very low leaching rate, and, thus, represent a robust and competitive technology for chemical manufacturing.

## 2. Supported liquid-phase catalysts based on organic solvents

Besides a few patents from the late 1930s on the use of supported Brønsted acids for olefin polymerizations [20], the idea to use a SLP for the immobilization of catalytically active solutions of transition metal compounds originated from independent reports of industrial research laboratories at Johnson Matthey [21] and Monsanto [22] in 1966. It was the same year Osborn *et al.* [23] reported on the preparation and catalytic properties of [RhCl(PPh_3_)_3_], which makes SLPs the oldest by-design approach to continuous-flow multiphasic homogeneous catalysis in the open literature. Ethyleneglycol solutions of RhCl_3_-hydrate were supported on porous silicates by wet impregnation with MeOH, and drying yielded free-flowing powders containing the ethyleneglycol catalyst solution in the pores of the support material [21]. The gas-phase isomerization of pentenes was studied as model reaction. Despite good initial activity, a progressive deactivation was detected in pulsed continuous-flow mode. In their conclusion the authors stated that ‘… the application of this method to other possibly more amenable and commercially important systems will readily be conceived’, and a patent was filed the same year [21]. The researchers at Monsanto [22] modelled the kinetic behaviour of SLP catalysts in detail, and derived mathematical functions describing the diffusion resistance of gaseous substrates to the homogeneous catalysts in the SLP. They found relations very similar to the classical pore diffusion limitation characteristically encountered in heterogeneous catalysis, where the extent of the pore diffusion limited regime of a given reaction is determined by the pore structure of the solid (Thiele modulus [24]). Importantly, for SLP catalysts, this regime was found to be a function of the liquid loading (defined either as wt% liquid or as pore filling = liquid volume/total pore volume). This prediction was verified experimentally for the continuous gas-phase hydroformylation of propylene with [RhCl(PPh_3_)_2_CO] immobilized in butylbenzylphthalate on silica gel [25]. The decrease in catalyst performance above intermediate levels of loadings was interpreted as the onset of diffusion limitation through the SLP. In these experiments, a pre-saturator bed (butylbenzylphthalate on silica gel) and an adsorber bed (dry silica gel) were placed before and after the SLP catalyst, respectively. From post-reaction gravimetric analysis it was concluded that little to no exchange of SLP between the beds occurred under reaction conditions, confirming effective retention of catalyst and SLP [25]. In 1973, Rinker and co-workers [26] proposed a simple experimental method for locating the optimum pore filing of SLP catalysts for minimum mass transfer limitation: isobaric gas-uptake curves of non-reactive SLP materials with various liquid loadings yields diffusion rates as a function of pore filling.

After these early reports many industrially employed solid catalysts were re-examined, and some were found to be SLP-type systems under reaction conditions. An extensive review summarizing the state of the art of 1978 [27] also includes early examples of ‘molten salt SLP catalysts’ [28]. Under consideration of different attractive interactions between solids and liquids (capillary and adhesive forces), the microscopic distribution of the SLP on the surface of the porous support materials was discussed in the same paper. In analogy to chromatography, a wetting model was proposed and an analytical function for the maximum radius of liquid-filled pores was derived on the basis of equal chemical potential of surface film and pore filling [29]. As suggested earlier, the diffusion limitation of SLP was confirmed to be a function of liquid loading also in these models.

In a series of seminal papers [30–35], Scholten and co-workers [30] studied the gas-phase hydroformylation of propylene with [HRh(PPh_3_)_3_CO] in SLP in great detail. The supported catalyst materials were analysed by multiple techniques including differential scanning calorimetry (DSC), microscopy, porosimetry, gas adsorption and IR spectroscopy. Intrinsic reaction rates, activation energies and diffusion effects were experimentally measured and fitted by theory [34]. Different substrates, additives and ligands (including phosphines, arsines and amines) were screened [33], the respective liquid loading and reaction conditions optimized [31], and support surfaces modified [32]. Under optimized conditions, short activation periods and suppressed formation of aldol-condensation side products could be achieved: over 800 h of stable continuous operation at TOFs exceeding 2000 h^−1^ with 99.5% selectivity to aldehydes at *l*/*b* ratios of up to 8.8 were achieved with neat molten PPh_3_ as the SLP on mesoporous silica.

Haumann and Wasserscheid recently reported continuous-flow hydroformylation of ethene and 1-butene in which a rhodium catalyst modified with sulfoxantphos or a diphosphite ligand was directly physisorbed on silica. During the reaction, high-boiling aldol-condensation side products filled the pores of the support acting as a solvent for the homogeneous catalyst as revealed by the analysis of the spent catalyst. The catalyst system based on the diphosphite ligand led to a conversion of 1-butene ranging from 47.3% to 41.5% corresponding to a TOF of 751 to 659 h^−1^ over 77 hours-on-stream [36,37]. A long-term run (approx. 1000 h on-stream) showed quite stable catalyst performances, however, not reaching the excellent values registered with an identical catalyst system including an IL film (*vide infra* §4c).

Hydrogenations of ketones and aldehydes with homogeneous ruthenium complexes in SLP have also been reported [38]. Even high-boiling substrates such as cycloheptanone could be passed over the SLP bed with H_2_ as strip-gas. The transport phenomena occurring in porous SLP catalysts were investigated in detail by Rinker and co-workers [39–41], who derived more elaborate models than initially proposed and also verified the predictions experimentally.

Engineering aspects of SLP catalysts in continuous-flow mode and the influence of various reactor configurations on the stability of such systems were studied by Stegmueller & Hesse [42]. It was shown that pre-saturation of the mobile gas phase with the supported solvent at reaction temperature may compensate for progressive solvent loss of the SLP [43], and thus prevent deactivation through catalyst precipitation. Over 700 h of stable propene hydroformylation were demonstrated in a fluidized bed reactor [44]. The optimum loading phenomenon was re-investigated [45], and the influence of the pore structure [46] and pore size distribution on the optimum value was modelled by three-dimensional simulations [47]. Using again more sophisticated models, recent simulations distinguished uniform from non-uniform film distributions [48,49] which were successfully validated against earlier literature results. In 2003, it was demonstrated that the progressive solvent loss encountered in long-term continuous gas-phase application may be compensated also by use of reversed-flow techniques [50].

## 3. Supported aqueous phase catalysts

In 1989, the concept of SLP was extended to supported aqueous phase (SAP) catalysis [51]. While the early SLP catalysts relied on a difference in volatility as separation barrier, retention of SAP catalysts was based on polarity differences. Thereby, conversion of liquid substrates became accessible, provided they were of sufficient hydrophobicity. In this respect, the large surface area and short diffusion pathways appeared particularly advantageous over bulk aqueous-organic biphasic systems.

The hydroformylation of oleyl alcohol with TPPTS-modified rhodium complexes in SAP on porous glasses with hydrophilic surfaces was demonstrated batch-wise with non-detectable rhodium leaching [52]. It was proposed that the catalysis proceeded just at the aqueous-organic interface. A strong dependence of the activity, selectivity and stability of the SAP catalysts in the hydroformylation of various substrates on the water loading used (added via vapour condensation after impregnation of the neat catalyst complex on the support) was noted, with an optimum water content at 4–12 wt% [52]. On the basis of detailed NMR analysis, this particular system was shortly after suggested not to be a genuine SAP catalyst, but rather a surface-adsorbed catalyst operating in the organic substrate phase [53]. Hydrogen bonding of the surface silanols to the sulfonate groups in a minimal aqueous film was proposed as more realistic description. This proposition was later verified independently for similar complexes [54], and developed further into an immobilization strategy of its own [55]. The altered selectivity of the aqueous system was ascribed to water-mediated hydrogen bonding between the sulfonato-groups of neighbouring TPPTS on Rh, forming a weakly associated multidentate ligand scaffold [56]. The rhodium-catalysed hydroformylation of propene in SAP was recently re-investigated with regard to different ligands, ligand to metal ratios, support materials and reaction conditions [57].

SAP catalysis has also been used for the selective hydrogenation of α,β-unsaturated aldehydes with Ru catalysts on silica [58]. Asymmetric C=C hydrogenation using a sulfonated Ru-BINAP complex was shown to proceed with up to 70% *ee* in SAP [59]. The same system was subsequently transferred ‘back’ to SLP using anhydrous ethyleneglycol instead of water, which increased enantioselectivity to 96% *ee*, the same value achieved in homogeneous solution in the absence of support [60]. For the hydroformylation of acrylic acid esters with Rh-SAP catalysts, higher activities than in bulk biphasic systems were found [61]. Using an optimum loading of 37 wt%, the TOFs of the SAP catalyst reached values as high as 2370 h^−1^, roughly one order of magnitude higher than under bulk biphasic conditions.

Wacker oxidation of liquid olefins with O_2_ and Pd/Cu catalysts in SAP has also been demonstrated successfully [62]. The lower activity compared to the bulk aqueous system was explained on basis of restricted mobility of the two metal catalysts, which need to interact during the catalytic cycle. Pd-catalysed allylic alkylations have also been studied in SAP [63]. Catalyst leaching and Pd black formation were claimed to be suppressed in SAP, but decreased activities were still observed during batch-wise recycling.

Arai and co-workers [64] reported an interesting example of cascade catalysis by multiple organometallic complexes in SLP/SAP, which proved incompatible in homogeneous solution. The simultaneous selective hydrogenation of two different substrates in a mixture with two SAP catalysts inside one reactor was demonstrated as well as a catalytic sequence of Heck coupling and hydroformylation with two different SAP catalysts. However, only one recycling experiment was conducted for the hydrogenation system, and none for the SLP cascade.

## 4. Supported ionic liquid-phase catalysts

With the advent of ILs as designer solvents for organometallic catalysis in the late 1990s [65–67], the extension of the SLP concept to supported ionic liquid-phase (SILP) systems became manifest. The advantage over organic SLP or SAP systems was thought to be that SILP would benefit from both polarity and volatility barriers, and would thus permit liquid as well as gas-phase applications with a variety of catalysts [68]. In general, the nature of the anion of the IL has a strong impact on the catalytic outcome as most of the organometallic catalysts are cationic species and low coordinating anions are usually employed to preserve the original catalytic performances [69,70]. On the other hand, imidazolium-based IL may serve as reservoir for the formation of NHC ligands either upon deprotonation or oxidative addition to zero-valent metals [71,72]. The formation of metal–NHC complexes have a special relevance in Pd-catalysed transformations like Suzuki and Mizoroki–Heck reactions [73,74].

### (a) Synthetic methods

Different synthetic strategies to SILP-type materials have been developed, which can be categorized into three different methods:

(I) deposition of IL/catalyst solutions on the surface of a porous support via wet impregnation (*physisorbed SILP*);
(II) chemical functionalization of a surface with a component of the IL (*chemically anchored SILP*);
(III) sol–gel synthesis of a porous solid in the presence of the IL/catalyst solution (*ionogels*).

Method (I) is the classical approach developed for SLP materials, and represents the most versatile strategy. The preparation procedure is straightforward, well reproducible, scalable and a wide range of different components can efficiently be combined and screened [75]. The more sophisticated method (II) may enhance the affinity (and thus retention) of SILP and catalyst on the support, and extends the range of support materials to non-porous structures such as organic polymers, metal fibres or carbon nanotubes (CNTs) [76]. It is typically used in conjunction with method (I) to introduce more IL and the catalyst. Capillary forces are of little relevance to method (II), and high surface coverage is typically achieved. Method (III), a material science approach, had been developed independently from SLP methodology in the early 1990s [77]. It requires a robust (pre)catalyst that survives the material synthesis procedure (sol–gel chemistry [78,79] of inorganic oxides, metal-organic frameworks, etc.), and poses more difficulties on post-synthetic characterization. The SILP containing the catalyst is encapsulated in inner pores of the solid by various degrees, depending on the structure of the material. Although examples of successful application in catalysis have been reported [80–87], these materials will not be discussed further here as the micro-environment around the catalyst is distinct from the film-like situation in SLP systems.

### (b) Characteristics

Owing to very high surface to volume ratios, fluids confined in nanospaces experience changes in some of their physical properties (Gibbs–Thompson effect) [88]. Upon deposition on dehydroxylated porous hydrophilic silicates, carefully dried hydrophobic ILs experienced melting point depressions of over 30°C, with an inverse relationship between pore size and melting point depression [89]. Comparison showed that the effect was even stronger for these ILs than for water, which may be taken as indication for specific IL-surface interactions atop physical confinement effects. These phenomena have been analysed further by diffuse reflectance IR (DRIFTS), DSC and polarized optical microscopy (POM) varying both support pore sizes and IL loadings, in concert leading to the proposal of an approximately 2 nm thick contact layer in which surface interactions predominate [90].

The thermal stability of BMIM PF_6_ has also been found to be influenced by the presence of silica [91]. Thermogravimetric analysis (TGA) and high vacuum distillation experiments showed that thermal decomposition of the IL was lowered by more than 100°C when deposited on dehydroxylated silica or alumina as compared to the bulk IL. These effects have been systematically studied for a variety of imidazolium SILPs on various oxide surfaces at a fixed loading of 17 wt% (i.e. varying pore filling and/or film thickness) [92]. The interplay of cation-anion ion pairing (H-bonding as followed by ATR-FTIR) versus surface acidity (zeta potentials in aqueous suspension) was found to correlate with the decrease in thermal stability of the IL on the surface, and a model was derived that may serve to predict thermal stabilities of new SILPs.

Catalytically active SILP materials containing [Pd(DPPF)(CF_3_CO_2_)_2_] and CF_3_SO_3_H in imidazolium ILs on fumed amorphous silica have been analysed for component interactions by multiple techniques [93]. From N_2_ adsorption isotherms and TEM images it was concluded that pores up to 9 nm radius were flooded by the IL, whereas larger pores were only surface-covered. By line-width analysis of SS-MAS NMR spectra it was shown that the mobility of the imidazolium cations and the Pd complex were reduced in the SILP material. The formation of ordered solvent cages around the organometallic complex was suggested as possible explanation for this observation at the high molar ratio of Pd to IL (1 : 25–33) used in these experiments.

The transport properties of hydrophilic and hydrophobic solutes through SILP membranes with hydrophobic ILs has also been studied [94]. Furthermore, it was attempted to quantify the degree of polarity change that imidazolium ILs undergo when covalently immobilized on different polymers [95]. By comparing π* values [96] of the polymer-SILPs with different organic solvents and the blank polymers, increased micropolarity of the SILP materials was inferred. However, direct comparison of IL in bulk form and as SILP is currently not available.

**Figure 3.**
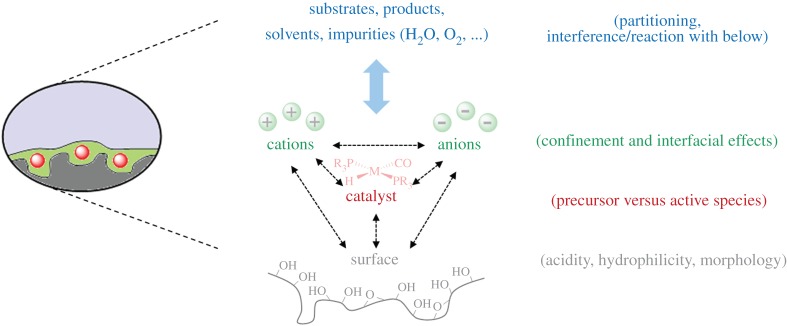
Interactions to be considered in understanding and tuning functional SILP catalyst materials.

As a result of multiple intermolecular forces (electrostatic attraction, H-bonding, *π* and hydrophobic interactions), ILs are highly ordered solvents [97] which often display specific interactions with solutes [98–101]. As to which degree the bulk solvent properties of ILs are altered upon their deposition on the surface of a support is a challenging question which, despite much effort, has not yet been fully resolved [102]. So far, mainly model systems on well-defined surfaces have been investigated by e.g. X-ray reflectivity [103], sum-frequency vibrational spectroscopy [104–106], electrical impedance spectroscopy (EIS) [107] and X-ray photoelectron spectroscopy (XPS) [108,109], all under analytic conditions, respectively. The difference to reactive systems is highlighted by findings on surface rearrangements of ILs containing polar transition metal complexes [110]. The use of XPS for studying IL interfaces, including some reactive systems, has been reviewed [111]. From most spectroscopic studies a different orientation of the IL layer in direct contact with the surface and the upper layers was generally deduced (as mentioned above [90]). Strong, directed hydrogen bonding of water in wet ILs to both surface silanols and NTf_2_ anions [112] has been observed by sum-frequency vibrational spectroscopy [104]. A low-temperature, ultra-high vacuum IR study of [Ru(CO)_3_Cl_2_]_2_ in thin films of BMIM NTf_2_ on alumina introduced by physical vapour deposition showed that multi-component systems can be surface-analysed under carefully chosen conditions [113]. However, implications for catalytic systems under reaction conditions are still difficult to rationalize from these data. In one notable example, an *in situ* *para*-hydrogen induced polarization (PHIP) NMR study on propene hydrogenation with bis-phosphine rhodium complexes in SILP suggested a relocation of the catalyst from the IL–gas interface to the IL-support interface under continuous-flow conditions [114]. A DRIFTS investigation of EMIM NTf_2_ on dehydroxylated silica under strictly anhydrous conditions showed that hydrogen bonding between surface silanols and the anion dominate the interfacial interactions, with the cation showing no changes as compared to the bulk IL [115]. Although the cation did not engage in interactions with the silica surface in the bare SILP, the same study found evidence for proton scrambling between surface silanols and the C2 hydrogen of the imidazolium cation under reaction conditions as evidenced by isotope labelling experiments, highlighting the importance to investigate reactive systems in addition to model studies. Figure 3 illustrates the challenges in understanding SILP catalysts arising from the numerous interactions that occur in these multi-component materials, in particular under sustained continuous-flow conditions where trace impurities in the feed may accumulate in the SILP over time.

Despite their relative young history, SILP-type materials have already found applications in many different areas [116–122]. Engineering applications such as gas separation processes [123,124] or selective absorption techniques [125] will not be considered in the present contribution. Selected examples of catalytic applications will be briefly discussed, with the focus lying on molecularly defined organometallic catalysts. Therefore, biocatalysis [95,126], organocatalysis [127], metal nanoparticles [128] and heterogeneous catalysts with IL-coatings [129] will not be considered. Polymer-supported ILs are included only when of relevance to molecular transition metal catalysis.

### (c) Gas-phase applications

The first intentional organometallic SILP catalysts were prepared by Carlin and co-workers in 1998 [130]. [Rh(nbd)(PPh_3_)_2_]^+^ in different imidazolium ILs was deposited on porous polyvinylfluoride by wet impregnation. The materials were used for the continuous hydrogenation of propylene and ethylene, with olefins and hydrogen fed from different sides of the membrane, respectively. Various IL anions were screened, and activity trends could be related to gas solubilities. Dimerization of ethylene with a [NiCl_2_(Pcy_3_)]_2_/EtAlCl_2_ catalyst in chloroaluminate ILs on polyethersulfone membranes was also reported in the same year [131].

Continuous-flow rhodium-catalysed hydroformylation of propene with SILP catalysts based on rhodium-phosphine catalysts was reported by Wasserscheid, Riisager, Fehrmann and co-workers [132,133]. Different ligands, ILs and IL-loadings on silica were screened for optimum activity and stability. At 100°C, the best system at low IL loadings of 11 wt% (8% pore filling) based on the sulfoxantphos ligand achieved a TOF of 37 h^−1^ with linear/branched ratios up to 23. After 4 h of operation deactivation set in regardless which IL or loading was used. The system was developed further and improved by optimizing the pre-treatment of the support surface (dehydroxylation at 500°C) [134,135]. Through solid-state ^31^P NMR, it could be shown that some of the excess of free phosphine got protonated by the silanols groups of the support. In more detailed investigations, the Wasserscheid group [136] improved the materials systematically to reach stabilities over 200 h at TOFs around 100 h^−1^, and transferred the process to gradient-free loop reactors to facilitate kinetic analyses. A breakthrough was reached by Haumann and Wasserscheid when introducing a bulky biphephos derivative bearing benzopinacols substituents as the ligand. The SILP material based on Rh/diphosphite-EMIM NTf_2_ and dehydroxylated silica (*α*=0.1) allowed for continuous-flow olefin isomerizing hydroformylation of a diluted industrial feedstock containing isobutene (43.1%), 1-butene (25.6%), *trans*-2-butene (9.1%), *cis*-2-butene (7.0%), non-reactive butanes (14.9%) and 1,3-butadiene (0.3%) with a selectivity of more than 99% towards *n*-pentanal under all applied conditions. Using a dried gas feed and adding an acid scavenger, the catalyst activity could be retained for more than 800 h, achieving TONs of approximately 350 000 and an average TOF of 410 h^−1^. By increasing the temperature from 100°C to 120°C, an enhanced TOF of up to 3600 h^−1^ and a STY of up to 850 kg_*n*−*pentanal*_ m^−3^ h^−1^ could be obtained and maintained for at least 10 h on stream [137,138]. The same authors also showed that the feed for the hydroformylation process can be generated *in situ* by dehydrogenating butane over a heterogeneous Cr/Al_2_O_3_ catalyst. Thus, combining dehydrogenation followed with isomerizing hydroformylation, a two-step conversion of butane directly to *n*-pentanal was achieved [139].

Continuous methanol carbonylation with MeI co-feed using a Monsanto-type catalyst in SILP was also reported [140]. Moderate rates and selectivities were obtained, and stable operation was only possible for the first 90 min. More recently, the Wasserscheid group [141] reported continuous oxycarbonylation of MeOH to dimethylcarbonate catalysed by simple copper halides in SILP. At 110°C activities were in the order of a few turnovers per hour but could be improved by optimization of the SILP components to reach 40 h of stable operation, yielding approximately 600 TON.

Kiwi-Minsker and co-workers [142] reported on rhodium-based hydrogenation catalysts in SILP on microstructured support materials consisting of plates of sintered metal fibres coated with carbon nanofibres. TOFs of 250 h^−1^ with stability over 6 h were achieved in the continuous hydrogenation of 1,3-cyclohexadiene. The stability of catalyst performance was tentatively ascribed to the high heat conductivity of the support, suppressing hot spot formation during the exothermic hydrogenation reaction.

Low-temperature water-gas-shift (WGS) catalysis with SILP materials was reported by Wasserscheid and co-workers [143]. The initial catalyst systems showed limited activity and stability, but could be greatly improved through tuning of the support basicity and use of preformed Ru-catalysts. Stable operation over 100 h with good activity for a molecular WGS catalyst could eventually be achieved at temperatures as low as 120°C [144].

Continuous-flow hydroaminomethylation of ethylene with diethylamine to give diethyl- propylamine, catalysed a Rh-Xantphos system in various ILs on different supports has been studied [145]. Best results were achieved in MMMIM NTf_2_ on a porous carbon support (PBSAC) at loadings of *α*=0.1. Selectivity to diethylpropylamine was 99%, effectively suppressing aldol side reactions. A slight decrease in activity occurred over 400 h of operation, nevertheless reaching 115 000 TON.

Gas-phase ethylene dimerization to 2-butenes catalysed by cationic Ni(II) complexes bearing chelating P−O ligands in EMIM FAP on dehydroxylated silica gel was also studied in continuous flow [146]. Initially, high conversion and selectivity were observed in the first 10 h on stream after which rapid deactivation due to progressive thermal runaway along the fixed-bed reactor occurred. Variation of IL loading, catalyst loading and reaction temperature improved the heat management, which together with a more stable Ni complex afforded lifetimes of more than 200 h reaching 97 000 TON. Utilization of a purified ethylene feed and application of a fluidized bed reactor yielded further improvement in catalyst stability [147].

One noteable example from the Wasserscheid group demonstrated continuous gas-phase asymmetric C=O hydrogenation of the high-boiling substrate methyl-acetoacetate (b.p. 170°C/70 mmHg) using a chiral Ru-phosphine–phospholane complex in SILP. Under optimized conditions (substrate delivery as a methanol solution; carrier gas He; *T*=105°C) after an induction period of 35 h, stable conversion around 70% and an enantioselectivity of 75–80% *ee* could be realized for 70 hours-on-stream in a tubular reactor. High IL loading (*α*=0.8) of the support (silica 30) was necessary to maintain stable performances, and a TON of 2500 was achieved in this experiment [148]. Under similar conditions the asymmetric hydrogenation of the more challenging methyl pyruvate with a Ru-BINAP SILP catalyst resulted in lower stability and *ee*’s (26–30%) [149].

### (d) Liquid-phase applications

Hölderich described Lewis-acidic ILs with chloroaluminate anions deposited on various porous oxide supports, which were used as catalysts for Friedel–Crafts alkylations of aromatic compounds. Covalent surface attachment via condensation of a siloxane functionality in the alkyl chain of the cation was used (method (II)) [150]. The range of materials was broadened to different Lewis-acidic anions and more catalytic reactions were screened [151]. However, liberation of HCl from reaction of the silanols groups with the chloroaluminates led to partial destruction of the oxide support. Continuous liquid-phase application showed limited stability with obvious deactivation within a few hours [152]. Similar materials bearing chlorostannate ILs were used by the Landau group [153] as catalyst for the Prins condensation of ^iso^butene with formaldehyde, showing moderate stability in repetitive batch experiments.

Friedel–Crafts isopropylation of cumene catalysed by Lewis-acidic SILP materials, similar to the ones of Hölderich [151], was compared under liquid-phase and gas-phase conditions [154]. Besides different regioselectivities of alkylation, the SILP materials were more active than the catalyst in bulk-IL biphasic application. The loading was varied and the support surface pre-treated with the chloroauminate IL in CH_2_Cl_2_ to activate the surface for enhanced acidity. Although AlCl_3_ leaching in the per cent range was observed in liquid phase, the materials could be recycled four times batch-wise without alteration of activity, but changing selectivity. In continuous gas-phase application, the materials could be used for more than 200 h on stream with stable performance [155]. The quality of the reagents with respect to high-boiling impurities and residual water content was found to be of crucial importance.

Mehnert reported on solution-phase applications of SILP catalysts based on Rh-catalysed olefin hydroformylation [156] and hydrogenation [157]. Both chemically anchored imidazolium fragments (method (II)) as well as purely physisorbed SILP (method (I)) were prepared using BMIM PF_6_ and BMIM BF_4_. For the hydroformylation of 1-hexene, a 10 : 1 mixture of TPPTS and [Rh(acac)(CO)_2_] was used as catalyst with 25 wt% IL-loading and applied in neat liquid substrate. At 100°C, the SILP system achieved nearly three times the rate of the unsupported bulk biphasic system (TOF=3900 h^−1^ versus 1380 h^−1^), which was attributed to enhanced catalyst accessibility in SILP. However, at conversions more than 50% leaching of IL into the liquid product phase entrained up to 2% of the catalyst per run. The hydrogenation SILP catalyst containing [Rh(nbd)(PPh_3_)_2_] PF_6_ in BMIM PF_6_ on silica did not suffer from such strong depletion effects, due to the cationic nature of the active catalyst and because the liquid substrate/product phase remains unpolar throughout the reaction. No metal could be detected in the product phase (less than 0.03 ppm), and the SILP catalyst could be recycled for at least 18 times without apparent deactivation in batch mode. Electron microscopy confirmed the absence of rhodium clusters more than 6 Å, and variation of hydrogen pressure revealed first-order kinetics in accordance with molecular catalysis in the SILP.

Serp reported on functionalized CNT as tailored support materials for hydrogenation catalysts in SILP [158]. Post-synthetic grafting of imidazolium fragments via amide linkers on carboxylate-CNT yielded structured supports, which were coated with BMIM PF_6_ containing [Rh(nbd)(PPh_3_)_2_] PF_6_ at various loadings (method (II)). The materials were analysed by different techniques and shown to afford much increased reaction rates (TOF 2880 h^−1^) in the hydrogenation of 1-hexene as compared to the same catalyst in SILP on oxidic support materials such as silica, titania, zirconia or alumina, and also activated carbon. The high thermal conductivity as well as the open channel structures of the CNT was used as explanation for this effect. The materials were recycled five times without loss of activity and undetectable rhodium leaching into the organic phase.

Ring-closing metathesis and cross-metathesis catalysed by Grubbs’ catalyst in HMIM PF_6_ on silica was reported by Hagiwara *et al.* [159]. Six recycling runs with decreasing activity were demonstrated. The materials were later used for the synthesis of macrocyclic lactones [160]. Ring-closing metathesis has also been performed with an imidazolium-tagged Grubbs’ catalyst in BMIM PF_6_ on a polyimide nano-membrane [161]. Stepwise catalysis in toluene and filtration showed pronounced deactivation in the third cycle, but catalyst leaching or IL cross contamination was not quantified.

Catalytic hydroamination of phenylacetylene with rhodium, palladium, copper and zinc complexes in SILP physisorbed on silylated diatomaceous earth (amorphous silica) using heptane as solvent was reported by Müller and co-workers [162]. Selectivities and activities of the complexes in SILP were found to be slightly higher than under homogeneous conditions, but no recycling experiments were reported.

Hagiwara *et al.* reported on palladium complexes immobilized in BMIM PF_6_ physisorbed on silica [163]. At 150°C, the materials were highly active in Heck-coupling reactions in dodecane as solvent and with tertiary amines as base. TOFs reached 8000 h^−1^ and batch-wise recycling was demonstrated for five runs. Decreasing activity could be restored by washing the SILP catalyst with aqueous NaOH. However, the formation of small amounts of homogeneous palladium species partitioning into the reaction medium under reaction conditions cannot be fully excluded.

Vankelecom and co-workers described the first chiral organometallic complexes in a SILP catalyst on a polar polymer as support [164]. Enantiomerically pure Ru-BINAP complexes in BMIM PF_6_ were used on poly(diallyldimethylammonium chloride) (method (II)) for the asymmetric C=O hydrogenation of methyl-acetoacetate. At 60°C, activities of the SILP catalysts were up to three times higher than the bulk IL-organic biphasic media with identical levels of enantioselectivity (97% *ee*). The catalysts were re-used once in batch mode, though catalyst retention or leaching was not quantified.

Müller and co-workers [165] reported on the enantioselective C=O hydrogenation of acetophenone with ruthenium- and rhodium-BINAP catalyst in SILP. While no enantioselectivity was observed for the reaction in MeOH, up to 74% *ee* were achieved with Rh-BINAP and K_2_CO_3_ in a tetra-alkylphosphonium carboxylate IL physisorbed on silica when used with hexane as solvent. Although no comparison of the same system in neat IL without support was reported, it was speculated that intensified substrate-catalyst interactions induced by the thin films of the SILP could be responsible for this effect. The hexane phase did not exhibit residual activity but recycling of the SILP catalysts was not performed.

Allylic substitution with Pd-catalysts in SILP on chitosan as support has been reported [166]. Drying of the support by scCO_2_ extraction yielded superior catalyst performance than freeze-drying from aqueous solutions. Performing catalysis in neat substrate, leaching issues could not be fully resolved (up to 9% per run), and stability was rather poor during batch-wise recycling experiments (complete deactivation from fifth cycle). Asymmetric versions were also demonstrated, but without recycling experiments.

Dioos & Jacobs reported on continuous liquid-phase application of chiral Cr-salen complexes in SILP physisorbed on silica [167]. Asymmetric ring opening of epoxides with TMSN_3_ was performed yielding good enantioselectivities in the range of 65–96% *ee*, reaching 314 TON. Catalyst leaching up to 1% was observed even with hexane as mobile phase.

Hardacre co-workers [168] reported on asymmetric Mukaiyama aldol reactions catalysed by bis(oxazoline)-copper complexes in SILP and compared the results to the non-supported IL system. In bulk EMIM NTf_2_, the Lewis-acid promoted condensation of methylpyruvate and phenyl-TMS-ethene was at least 60 times faster than in CH_2_Cl_2_ with complete retention of enantioselectivity (82% *ee*). However, hydrolysis of the TMS-ether resulted in about 10% lower chemoselectivities. Adsorbing the IL on either imidazolium-functionalized or plain silica, this side reaction was effectively suppressed, and the SILP catalyst combined high activity with good enantioselectivity when applied in a biphasic system with Et_2_O. While neutral copper catalysts leached out to 19%, an imidazolium-modified ligand afforded enhanced retention significantly. However, deactivation was still noticeable after the fifth recycling.

The above examples show that a wide variety of homogeneous catalytic systems, including highly enantioselective ones, are compatible with SILP-type environments and that there is scope for activity improvement due to short diffusion pathways and large interfacial areas. A clear limitation of their application with liquid phases, however, is the effectiveness of catalyst retention; non-volatile, highly functionalized substrates and products require the use of good solvents which then often dissolve some of the IL film and leach out the immobilized catalyst. While volatility discrimination has been shown to be an effective separation barrier for continuous gas-phase application of thermally robust SILP catalysts (see §4c), polarity discrimination alone is clearly less effective. Unfortunately, the thermal process window of gas-phase processes excludes stereoselective transformations of complex molecules, one of the key skills of molecular transition metal catalysis. A gas-like solvent with liquid-like solvation power at mild temperatures would extend the potential of SILP catalysts to continuous-flow catalysis for fine chemical and pharmaceutical production. As shown in the following, SCFs show great promise in fulfilling precisely this role.

### (e) Supported ionic liquid-phase catalysts and supercritical fluids

The SCFs combine the transport properties of gases with the solvent properties of liquids for a wide variety of organic molecules [169]. In comparison to liquids, they exhibit low viscosity and high diffusivity, and are fully miscible with other gases like H_2_, CO or O_2._ They are, however, unable to dissolve ILs [170] or other low-volatile liquids [171], and are very poor solvents for many organometallic catalysts [172]. Thus, they have the potential to capitalize on the respective advantages of gas and liquid-phase processing in combination with SILP systems. scCO_2_ in particular is attractive due to its mild critical conditions (*T*_c_=31°C), and has been shown to exert some interesting effects on ILs, including reductions of melting point, interfacial tension, bulk viscosity and increased gas solubility [173]. A number of examples have successfully exploited these properties in liquid/SCF biphasic systems [174]. For example, high long-term productivity and enantioselectivity could be achieved for the asymmetric hydrogenation of β-keto esters in our group immobilizing the Ru-BINAP catalyst in bulk IL and using scCO_2_ as mobile phase [175]. In contrast to the SILP/gas-phase system reported by Wasserscheid and co-workers [148], substrates of low volatility could be used at milder temperatures contributing at least partly to higher stability and enantioselectivity with scCO_2_ as flow medium. Despite this promising perspective, the combination of SLP systems with scCO_2_ has been achieved only recently.

Pagliaro and co-workers [176] described sol–gel entrapped imidazolium ILs containing perruthenate *in silica* (method (III)) as highly active catalysts for the selective aerobic oxidation of alcohols. Application with scCO_2_ allowed efficient oxidation of non-volatile alcohols at 75°C. Batch-wise recycling was attempted, but practical difficulties of material loss prevented more than three repetitive runs.

**Figure 4.**
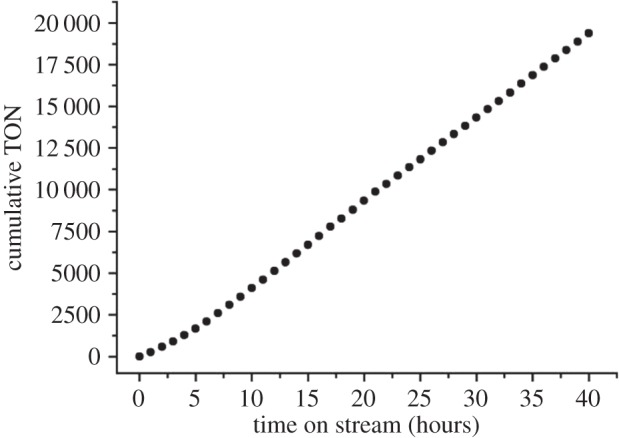
1-Octene conversion to aldehydes using SILP catalysts with continuous CO_2_ flow [177].

Cole-Hamilton and co-workers was the first to successfully apply organometallic complexes in SILP for continuous-flow catalysis with scCO_2_ [177]. The hydroformylation of 1-octene with Rh-TPPMS complexes in OMIM NTf_2_ on dehydroxylated silica (method (I)) proceeded with even higher rates than in bulk-IL/scCO_2_ media, and the system was very stable for at least 40 h (figure 4). 100°C reaction temperature, 21°C below the substrate boiling point, was sufficient by the use of CO_2_, and Rh-leaching levels were as low as 0.5 ppm in the organic product fraction recovered from scCO_2_ by decompression.

Stability and response times of the system were suitable for on-stream parameter variations for more detailed investigations using statistical methods. By variation of IL-loading, substrate flow and syngas pressure it was shown that at low loadings (29 wt%) higher syngas pressures decreased the rate (in accordance with the intrinsic reaction kinetics), while at high loadings (44 wt%) higher syngas pressure increased the hydroformylation rate. In conjunction with phase behaviour observations it was concluded that the reaction proceeded best in an expanded liquid substrate phase rather than in a single supercritical phase, and the IL film thickness became gas limiting at high loadings [178].

**Figure 5.**
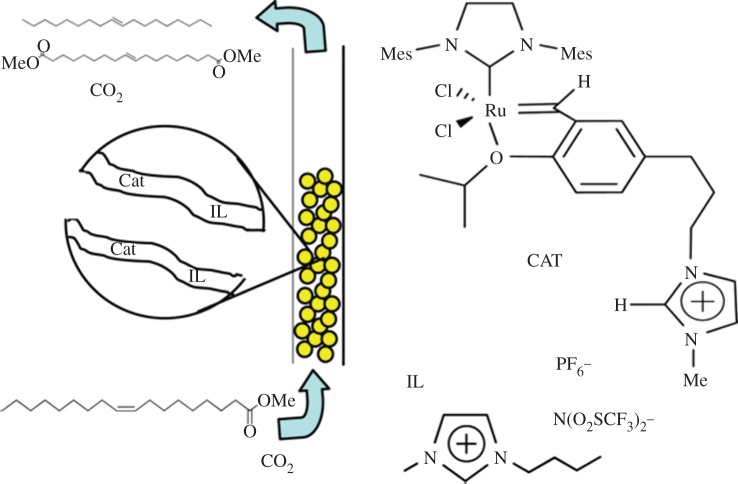
Conceptual visualization of the continuous SILP process for the Ru catalysed self-metathesis of methyl oleate with scCO_2_ [179].

Cole-Hamilton and co-workers [179] extended the approach to Ru-catalysed alkene metathesis using SILP catalysts in continuous-flow mode with compressed CO_2_ (figure 5). Self-metathesis of methyl oleate with an ion-tagged ruthenium catalyst in BMIM NTf_2_ on dehydroxylated silica (method (I)) proceeded with good rates for at least 9 h (TON>10 000), although slight loss of activity over time was observed. Ruthenium leaching levels were in the range of 10 ppm in the self-metathesis product. In the best case, 6 g of substrate per hour could be converted using a 9 ml reactor.

**Figure 6.**
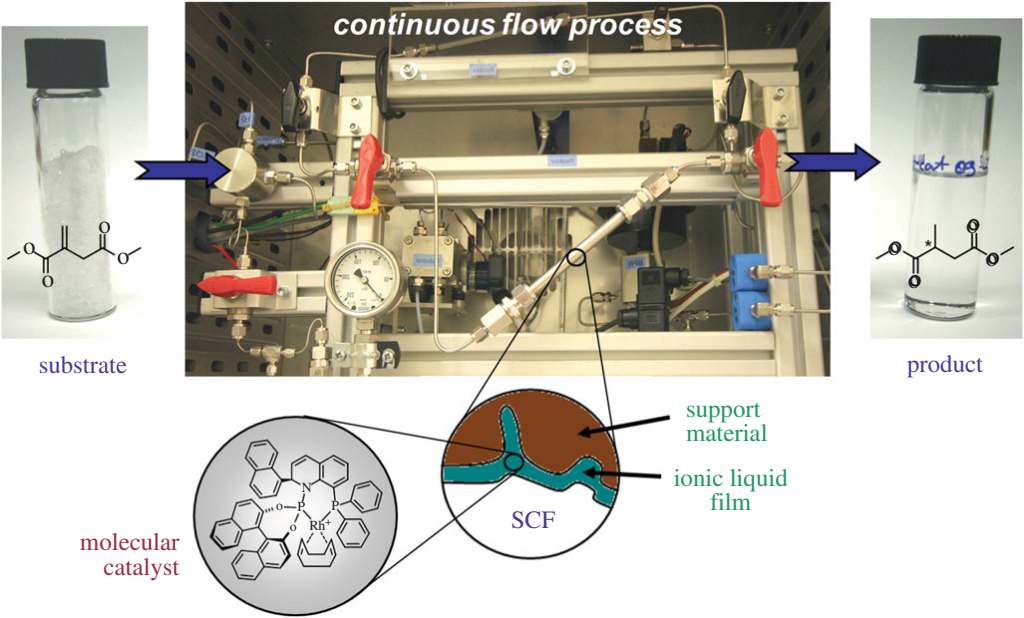
Continuous-flow asymmetric hydrogenation of dimethylitaconate with chiral Rh-QUINAPHOS catalysts in SILP with scCO_2_ [115].

The group of Leitner [180] developed a highly efficient example of enantioselective continuous-flow catalysis using chiral transition metal complexes in SILP. Rhodium catalysts comprising chiral ligands of the QUINAPHOS family were immobilized in SILP for the asymmetric C=C hydrogenation of dimethyl itaconate as prototypical example for a prochiral solid substrate (figure 6). The reaction was conducted in continuous-flow mode with scCO_2_ at 40°C using a flexible, fully automated set-up [181]. At quantitative conversion up to 99% *ee* were achieved, and 65 h of continuous operation were demonstrated, although at slightly reduced selectivity (70% *ee*) after 10 h on stream [115]. Remarkably, more than 100 000 TON for the chiral transition metal complex was reached. Productivities surpassed values of 100 kg product per gram rhodium, and the process operated at STY of 0.5 kg l^−1^ h^−1^. Rhodium leaching levels were below the detection limit of 1 ppm as judged by inductively coupled-optical emission spectroscopy (ICP-OES) of both the product fractions and the spent SILP catalyst. Fine-tuning of the support surface to avoid accumulation of trace water in the SILP turned out to be a crucial parameter for preventing progressive deactivation of the active species. Using either a water scavenger or a perfluoroalkyl-functionalized silica support increased catalyst stability to more than 80 h on stream to reach greater than 140 000 TON.

**Figure 7.**
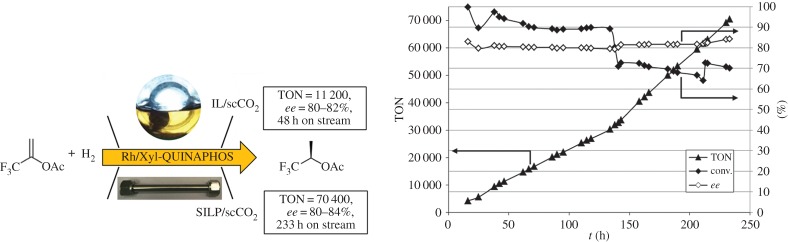
Continuous-flow asymmetric hydrogenation of 1-(trifluoromethyl)vinyl acetate: comparison of IL-scCO_2_ versus SILP-scCO_2_ immobilization strategies and long-term performanceof the SILP/scCO_2_ system (the variations in conversion reflect controlled variations of the substrate flow) [182].

Leitner and co-workers [182] compared the SILP-scCO_2_ immobilization strategy with the bulk IL-scCO_2_ approach in the asymmetric hydrogenation of the enol ester 1-(trifluoromethyl)vinyl acetate using the same molecular catalyst and IL (figure 7). Whereas the reaction in the liquid biphasic IL-SCF system was carried out in a continuously stirred tank reactor (CSTR), the solid biphasic SILP-scCO_2_ system could be implemented in a plug-flow reactor (PFR; see also figure 2). Using SILP as the catalyst matrix, the Rh/Xyl-QUINAPHOS catalyst afforded a sixfold higher productivity than that achieved with the IL-scCO_2_ system while no detectable metal contamination in the product stream was found for either system. In the SILP-scCO_2_ system the same catalyst led to remarkably stable performance during 233 h on stream with high single-pass conversions (90–70%), TON of 70, 400, and robust enantioselectivity (80–84% *ee*), which even slowly increased over time. Although Rh/Xyl-QUINAPHOS was the most active catalyst under batch conditions it also provided the best stability and highest productivity in continuous-flow application, highlighting the importance of long-term stability experiments for the identification of the most suitable catalytic system.

## 5. Conclusion and outlook

From the examples discussed in this contribution it becomes evident that SLPs represent a versatile and promising approach to using molecular catalysts in continuous flow. The concept of SLPs appears particularly attractive for ILs, because despite being good media for organometallic catalysis with extremely low volatility they suffer from high viscosity and cost. In SILP, the effective use of both solvent and catalyst in SILP catalysts is enhanced as compared to bulk-IL systems, because the catalyst is very close to a large interface in the ultra-thin film on the surface of the solid material. However, experience also shows that the combination of SLP materials with liquid flow media is bedevilled by progressive loss of the surface film through dissolution or physical abrasion under continuous operation.

The combination of SILP catalysts with compressible gases in the form of SCFs or expanded liquid phases offers particularly promising possibilities of broadening the window of applicable reactions and increasing overall process efficiency. Recovery of products from the mobile phase, free of any organic solvent, may be conveniently achieved by depressurisation or temperature swings [183]. Exciting combinations of selective product extraction [184] and continuous-flow cascade catalysis await to be realized on the basis of these developments. The *in situ* extraction of products from the catalyst surface may also be used to continuously re-adjust the equilibria of thermodynamically unfavourable reactions, thus overcoming equilibrium limitations [185]. Online catalyst analysis and reaction monitoring will greatly improve the understanding of these catalyst materials under reaction conditions and can enable self-regulating and even self-optimizing continuous-flow systems [186–192]. Another exciting possibility for asymmetric catalysis arises from recent progress in controlling the enantioselectivity of racemic or latent chiral complexes through their interaction with chiral ILs [193–195]. The IL can be tailor-made to carry acidic or basic functionalities, opening a molecular approach to multi-functional solid catalysts [196].

It is important to note that this is not restricted to organometallic catalysis, but enzymatic catalysis has also been efficiently carried out continuously in SILP/scCO_2_ and IL/scCO_2_ systems [68]. For example, the selective extraction properties of the SCF have been used to obtain enantiomerically pure products from kinetic resolution of alcohols with CALB using this technique [197]. In view of these exhilarating possibilities, it appears that the combination of SCFs with SLP-type catalysts are finally unlocking their full potential for fully integrated processes that selectively produce a single product in essentially pure form.
